# The “Radicular Tank”: A Novel Concept in Endodontics Achieved with the MEA Inverse Taper^®^ Technique

**DOI:** 10.3390/dj14030157

**Published:** 2026-03-10

**Authors:** Giovanni Messina, Gaia Bonandi, Marta Marchica, Marta Longo, Luigi Stagno d’Alcontres, Lusien Distefano, Antonino Cacioppo, Pier Edoardo Maltagliati, Calogero Bugea, Eugenio Pedullà, Elena Bardellini

**Affiliations:** 1 Independent Researcher, 25121 Brescia, Italy; giovanni.messina@brief-dental.com (G.M.); gaia.bonandi@hotmail.it (G.B.); 2 Independent Researcher, 90133 Palermo, Italy; martamarchica3@gmail.com; 3 Independent Researcher, 16126 Genova, Italy; martalongo91@hotmail.com; 4 Independent Researcher, 98121 Messina, Italy; lstagnodalcontres@gmail.com; 5 Independent Researcher, 95124 Catania, Italy; luisiendistefano@hotmail.com; 6 Departmento of Oral Sciences, University of Palermo, 90133 Palermo, Italy; cacioppoantonino@gmail.com; 7 Department of Surgical and Integrated Diagnostic Sciences, University of Genova, 16126 Genova, Italy; piermaltagliati@gmail.com; 8 Independent Researcher, 73014 Gallipoli, Italy; calogerobugea@yahoo.it; 9 Department of General Surgery and Medical-Surgical Specialties, University of Catania, 95124 Catania, Italy; eugeniopedulla@unict.it; 10 Department of Medical and Surgical Specialities, Radiological Sciences and Public Health, School of Pediatric Dentistry, University of Brescia, 25121 Brescia, Italy

**Keywords:** shaping systems, canal treatment, protocols, irrigants, dye retention

## Abstract

**Background:** Successful root canal treatment depends on the synergy between mechanical instrumentation and chemical disinfection. The internal canal geometry, particularly taper configuration, critically influences irrigant flow and penetration. Conventional taper designs tend to displace irrigants coronally, creating stagnation zones and limiting cleaning efficacy. The MEA Inverse Taper^®^ technique introduces a reversed taper geometry designed to retain irrigant within the canal during shaping, forming a fluid reservoir termed the Radicular Tank (RT). This proof-of-concept study aimed to experimentally demonstrate the formation of the RT generated by the MEA Inverse Taper^®^ design and to compare its qualitative hydrodynamic and shaping behavior with a conventional rotary system (MTWO). **Methods:** Standardized transparent canal models were instrumented using either the MEA Inverse Taper^®^ or MTWO sequence. A 1% methylene blue dye served as a visual tracer to assess potential intracanal retention at successive shaping stages. Standardized photographic documentation and digital image superimposition were used to evaluate residual dye retention, canal morphology, and taper variation. **Results:** The MEA Inverse Taper^®^ sequence maintained residual dye in the coronal and middle thirds, confirming the formation of the RT. Compared with MTWO, it produced a more conservative taper, minimized coronal and apical displacement of dye, and preserved canal curvature, removing less coronal dentin. **Conclusion:** The MEA Inverse Taper^®^ technique creates a qualitative dye-retention phenomenon (Radicular Tank) that allows continuous instrumentation within a visually persistent dye environment. This novel concept may support disinfection efficiency, alongside preserving dentin structure and reducing mechanical stress on rotary instruments, representing a potential advancement in endodontic shaping and irrigation protocols.

## 1. Introduction

Successful root canal treatment depends on the synergistic combination of mechanical instrumentation and chemical disinfection, a process referred to as chemo-mechanical preparation [1,2,3]. The primary goal of endodontic therapy is the complete removal of pulp tissue remnants and microorganisms from the root canal system, followed by three-dimensional obturation to prevent reinfection [4,5,6]. Among the numerous factors influencing clinical outcomes, the penetration and effectiveness of endodontic irrigants are considered crucial for achieving disinfection in anatomically complex and inaccessible canal areas [7,8,9,10,11].

Irrigants such as sodium hypochlorite and EDTA are responsible for tissue dissolution, bacterial elimination, and smear layer removal [12,13,14,15]. However, the efficacy of irrigant delivery and activation is profoundly influenced by the internal geometry of the prepared canal, particularly its taper configuration [13,14,15]. It has been demonstrated that increasing taper or modifying needle design does not necessarily improve irrigant penetration into the apical third, often resulting in stagnation areas (“dead zones”) and the formation of a coronal return vortex [15,16,17]. Moreover, needle design plays a critical role: side-vented needles tend to generate greater coronal backflow and reduced apical penetration compared with flat-ended designs, thereby limiting irrigant replacement and activation within the apical third [15,16,17]. These hydrodynamic limitations hinder effective disinfection and may compromise treatment success.

In recent years, the concept of minimally invasive endodontics has emphasized the preservation of pericervical dentin and the use of smaller tapers to enhance long-term tooth strength [18,19]. Nevertheless, conventional rotary instrumentation systems typically produce uniform or excessive taper, displacing irrigants both coronally and apically during shaping, thereby reducing their residence time within the canal [20,21]. Despite technological advances in activation systems and needle design, the dynamic behavior of irrigants under these conditions remains suboptimal [20,21,22,23].

To address these biomechanical and hydrodynamic limitations, the MEA Inverse Taper^®^ technique introduces a controlled inversion of taper geometry, allowing part of the dye volume to remain within the canal throughout the shaping process. This geometric inversion creates a visual dye-retention zone termed the Radicular Tank (RT), which acts as a temporary retention chamber for dye. In this study, the RT is defined as a localized intracanal dye-retention zone generated by controlled taper inversion. This retained dye volume functions analogously to the stagnation zones and coronal reservoirs described in hydrodynamic literature, but differs in that it is intentionally produced by instrument geometry rather than by unintentional flow limitations. It should be noted that, in this proof-of-concept study, the RT is identified through visual dye persistence rather than measurement of true irrigant volume, and is therefore interpreted as a qualitative hydrodynamic phenomenon rather than a quantitative fluid-retention system. The term is therefore used to describe an experimentally observable configuration of dye stability within the canal. The RT enables a true “instrumentation-in-bath” condition, where the rotary file operates continuously within an irrigant medium rather than in an intermittently replenished canal.

This configuration theoretically reduces debris extrusion, optimizes irrigant activation, and facilitates smoother file advancement with lower torsional stress [23,24].

While computational fluid dynamics (CFD) studies have analyzed irrigant motion in canals with standard taper geometries [20,23], no previous research has experimentally demonstrated a technique capable of maintaining an internal irrigant reservoir during mechanical shaping.

The MEA Inverse Taper^®^ sequence therefore represents a potentially paradigm shift in root canal preparation. The aim of this proof-of-concept study was to experimentally demonstrate the formation of the RT produced by the taper inversion geometry of the MEA Inverse Taper^®^ technique, and to compare its hydrodynamic and shaping characteristics with those of a conventional system (MTWO).

**Figure 1:**
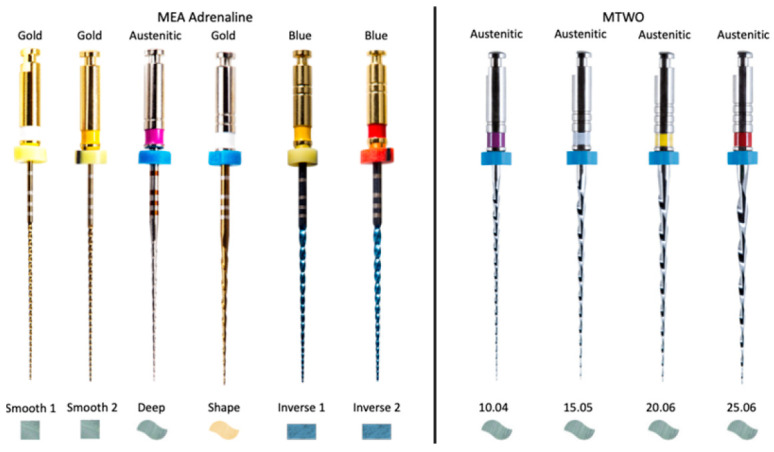
Cross-sections and NiTi alloy characteristics of the MEA Adrenaline (MEA Inverse Taper^®^) and MTWO instruments.

Conventional rotary systems such as MTWO are manufactured using Austenitic NiTi alloys and designed with a continuous taper that promotes efficient dentin removal and predictable shaping. Their S-shaped cross-section and progressive 0.06 taper are intended to enhance cutting efficiency while maintaining flexibility. In contrast, the MEA Inverse Taper^®^ system employs a hybrid NiTi metallurgy (austenitic–martensitic transition alloys) and a sequence featuring multiple cross-sectional geometries (square, S-shaped, and rectangular), as illustrated in Figure 1, specifically developed to reduce coronal dentin removal and alter irrigant dynamics. Unlike traditional designs, its geometry aims not only to preserve canal anatomy but also to modulate fluid behavior during shaping. These fundamental differences in taper design, instrument progression, and metallurgical processing provide the rationale for comparing the two systems within the present proof-of-concept study.

The working hypothesis was that the inverse taper technique would maintain irrigant retention within the canal during shaping, in contrast to the complete irrigant displacement observed in standard taper systems.

## 2. Materials and Methods

### 2.1. Study Design

This proof-of-concept experimental study was designed to evaluate the hydrodynamic and shaping behavior of the MEA Inverse Taper^®^ technique compared with a conventional rotary system (MTWO). The primary objective was to demonstrate, under standardized conditions, the formation of an irrigant reservoir—referred to as the RT—generated by the inverse taper geometry of the MEA system.

The materials and equipment used in the study are displayed in Table 1.

### 2.2. Overall Sample Size and Allocation

The overall sample size was *n* = 6 standardized transparent canal blocks, allocated to two experimental groups (MEA Inverse Taper^®^: *n* = 3; MTWO: *n* = 3). Each block was considered an independent experimental unit.

### 2.3. Specimen Preparation

Two commercially available endodontic systems were selected for comparison: MEA^®^ Adrenaline (MEA Inverse Taper^®^ sequence) and MTWO.

A total of six standardized transparent canal blocks were used for this proof-of-concept study, with three canals instrumented using the MEA Inverse Taper^®^ sequence and three canals instrumented using the MTWO sequence.

Standardized transparent endodontic training blocks (Dentsply-type, single canal, 16 mm working length, with a curvature located in the apical 5 mm) were used as experimental models. Each block simulated a single curved canal and was fixed in a custom-made acrylic support to ensure stable positioning during shaping and photography. The working length was established using an ISO #15 K-File (15.02) and verified with an EndoComfort Caliber.

### 2.4. Instrumentation Protocols

Root canal preparation was performed by a single experienced endodontist (G.M.), using a MotoSync Pro endodontic motor equipped with torque and auto-reverse control (TAT function).

Two instrumentation sequences were applied. The MEA^®^ Adrenaline (MEA Inverse Taper^®^) sequence consisted of: Deep 10.04 at 250 rpm and 1.2 N·cm torque; Shape 15.05, Inverse 1 20.04; Inverse 2 25.04 at 350 rpm and 1.2 N·cm torque. The MTWO sequence included: 10.04 at 250 rpm and 1.2 N·cm torque; followed by 15.05, 20.06, and 25.06 at 300 rpm and 1.2 N·cm torque.

All procedures were performed under 6× magnification using integrated LED illumination. Instrumentation was carried out in accordance with the manufacturer’s guidelines, using intermittent irrigation and a controlled pecking motion to simulate clinical shaping dynamics.

### 2.5. Irrigant Simulation and Visualization

To simulate irrigant behavior, a 1% aqueous methylene blue solution was used as a tracer fluid. After each shaping step, 2 mL of dye was injected into the canal using a 30-gauge side-vented irrigation needle placed 1 mm short of the working length.

Irrigation was performed after shaping with the Shape 15.05 instrument in the MEA Inverse Taper^®^ sequence and after shaping with the 15.05 instrument in the MTWO sequence.

This standardized protocol allowed visualization of residual dye and irrigant retention during the transition from intermediate to final shaping instruments. The presence, movement, and persistence of the dye were used as indicators of fluid dynamics and the canal’s capacity to retain irrigant throughout the shaping process.

### 2.6. Imaging and Data Acquisition

Photographic documentation was standardized using a Nikon D3000 digital camera mounted on a tripod. Camera position, focal length, and illumination intensity were kept constant for all acquisitions. To ensure precise alignment between pre- and post-instrumentation photographs, each plastic block was marked with reference notches on its coronal and apical surfaces. Images were acquired at three experimental stages: T_0_ (baseline), before instrumentation; T_1_ (intermediate), after shaping with Shape 15.05 (MEA) or 15.05 (MTWO); and T_2_ (final), after shaping with Inverse 2 25.04 (MEA) or 25.06 (MTWO). Each photograph included the entire canal and reference frame. Images were saved in RAW format to preserve color fidelity and allow quantitative post-processing.

### 2.7. Image Processing and Superimposition Analysis

Post-instrumentation images were imported into a digital editing suite for guided superimposition based on the reference notches. Residual dye retention and distribution were evaluated by measuring the area and intensity of methylene blue coloration in the coronal, middle, and apical thirds. Canal geometry was assessed by calculating diameters at predefined reference levels (D0–D11) according to manufacturer specifications and visual confirmation from the images. Comparative morphology between the MEA and MTWO sequences was determined by overlapping the final images to qualitatively and quantitatively evaluate residual dye, taper variation, and not instrumented canal walls. All analyses were independently performed by two calibrated examiners, and any discrepancies were resolved by consensus. Because the study aimed to demonstrate geometric feasibility rather than quantify fluid volume, residual dye distribution was used solely as a visual proxy for potential fluid-retention behavior.

### 2.8. Outcome Parameters

The primary outcome was the visual presence of residual dye used as a qualitative indicator of potential intracanal retention capacity (Radicular Tank) rather than a measurement of actual irrigant volume.

Secondary outcome included visual evidence of taper inversion and corresponding changes in canal diameter; comparison of irrigant displacement direction (apical *versus* coronal) between systems; and qualitative assessment of wall contact and potential debris extrusion zones.

### 2.9. Statistical Considerations

Given the exploratory and demonstrative nature of this proof-of-concept study, data were primarily analyzed descriptively. Quantitative observations—such as the relative area of dye retention—were expressed as proportions of the total canal surface, measured through pixel-based image segmentation. The study design focused on qualitative validation of the phenomenon, rather than inferential statistical analysis, in accordance with previous early-stage validation protocols for novel endodontic geometries [24]. Although the study was primarily qualitative, basic descriptive statistics (mean and range of dye-retention area obtained from pixel-based segmentation, calculated across all samples) were reported to provide additional clarity regarding the variability of the observations. No a priori sample size calculation or power analysis was performed because this study was designed as a proof-of-concept feasibility experiment with a primarily qualitative endpoint and descriptive analyses (no hypothesis testing). The chosen sample size (n = 3 per group) was intended to demonstrate reproducibility across independent standardized specimens and to provide an initial estimate of variability. Future confirmatory studies will be powered based on effect sizes derived from quantitative hydrodynamic outcomes.

## 3. Results

### 3.1. Qualitative Observations

Instrumentation with both systems was completed successfully in all plastic blocks without deformation or visible damage to the simulated canals. A clear difference in dye behavior was observed between the two systems throughout shaping.

**Figure 2:**
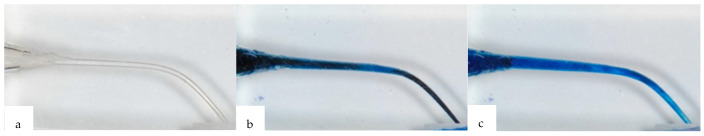
MEA Inverse Taper^®^ instrumentation sequence. (**a**) Baseline image (T_0_). (**b**) Intermediate stage (T_1_), after irrigation with methylene blue dye and shaping with the Shape 15.05. (**c**) Final stage (T_2_), after completion of the MEA Inverse Taper^®^ sequence, showing residual dye in the coronal third (Radicular Tank).

In the MEA Inverse Taper^®^ sequence, methylene blue dye persisted within the coronal and middle thirds throughout the entire instrumentation process. After shaping with the Shape 15.05 instrument (T_1_), dye was still visible along the canal walls, and upon completion of the sequence (T_2_), a distinct column of persistent dye remained in the coronal third, confirming the presence of the RT (Figure 2a–c).

**Figure 3:**
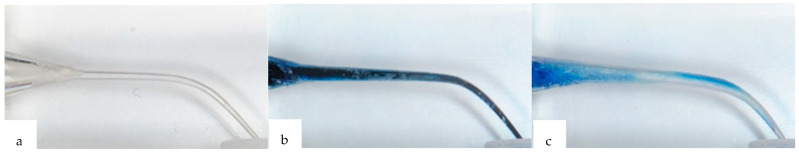
MTWO instrumentation sequence (control system) (**a**) Baseline image (T_0_). (**b**) Intermediate stage (T_1_), after irrigation with methylene blue dye and shaping with 15.05. (**c**) Final stage (T_2_), after shaping with 25.06, revealing complete displacement of dye and absence of irrigant retention.

In contrast, for the MTWO sequence, the dye was progressively displaced toward the canal orifice during shaping, leaving the apical and middle thirds devoid of dye after final preparation (Figure 3a–c).

### 3.2. Morphological Comparison

**Figure 4:**
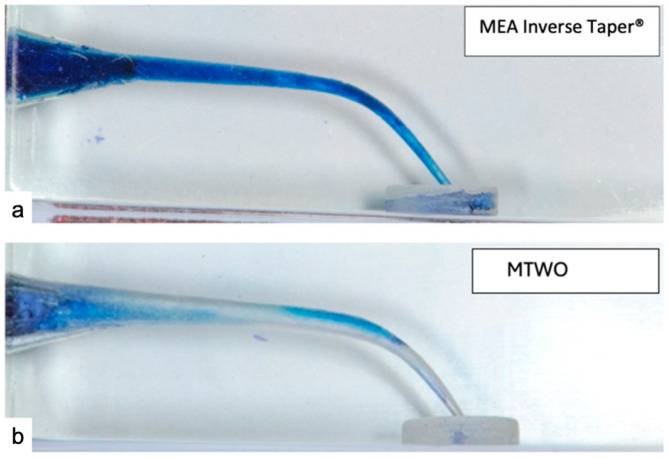
Final canal appearance after completion of shaping with MEA Inverse Taper^®^ and MTWO systems. (**a**) Canal prepared with the MEA Inverse Taper^®^ technique, showing residual irrigant retained in the coronal third. (**b**) Canal prepared with the MTWO system, showing complete removal of irrigant and full evacuation from the canal lumen.

The persistence of dye in the MEA Inverse Taper^®^ group confirmed the presence of an internal fluid retention zone—defined as the Radicular Tank—generated by the inverse taper geometry (Figure 3a). No similar phenomenon was detected in the MTWO group, where the dye was completely displaced coronally during file rotation, leaving an empty canal lumen (Figure 4b).

Moreover, the MEA Inverse Taper^®^ technique preserved the original canal curvature and resulted in less dentin removal in the coronal and pericervical regions compared to the MTWO system, which produced a more pronounced coronal enlargement (Figure 3).

### 3.3. Canal Geometry and Taper Inversion

Comparative visual and dimensional analyses revealed distinct morphological differences between the two systems.

In the MEA Inverse Taper^®^ sequence, canal shaping produced a controlled taper inversion beginning at level D6, where the canal diameter of the Inverse 1 (20.04) file (0.44 mm at D6) was slightly smaller than that of the preceding Shape 15.05 file (0.45 mm). Similarly, a second inversion occurred at D11, where the Inverse 2 (25.04) file exhibited a diameter of 0.69 mm, slightly below that of the Shape 15.05 (0.70 mm) (Table 2).

This double inversion generated a non-contact zone in the coronal and middle thirds, forming an internal cavity capable of retaining irrigant during file rotation. The final taper of the MEA Inverse Taper^®^ sequence averaged 4% up to D10 and 5% from D11 to D16, due to the earlier use of the Shape 15.05.

Conversely, the MTWO sequence exhibited a continuously increasing taper (average 6%), with complete wall contact and uniform dentin removal from coronal to apical thirds (Table 3).

This configuration provided no internal space for irrigant retention, leading to full fluid displacement and canal dehydration after shaping. Overall, these findings confirm that the MEA Inverse Taper^®^ geometry creates a unique double-taper configuration responsible for intracanal fluid stability.

**Figure 5:**
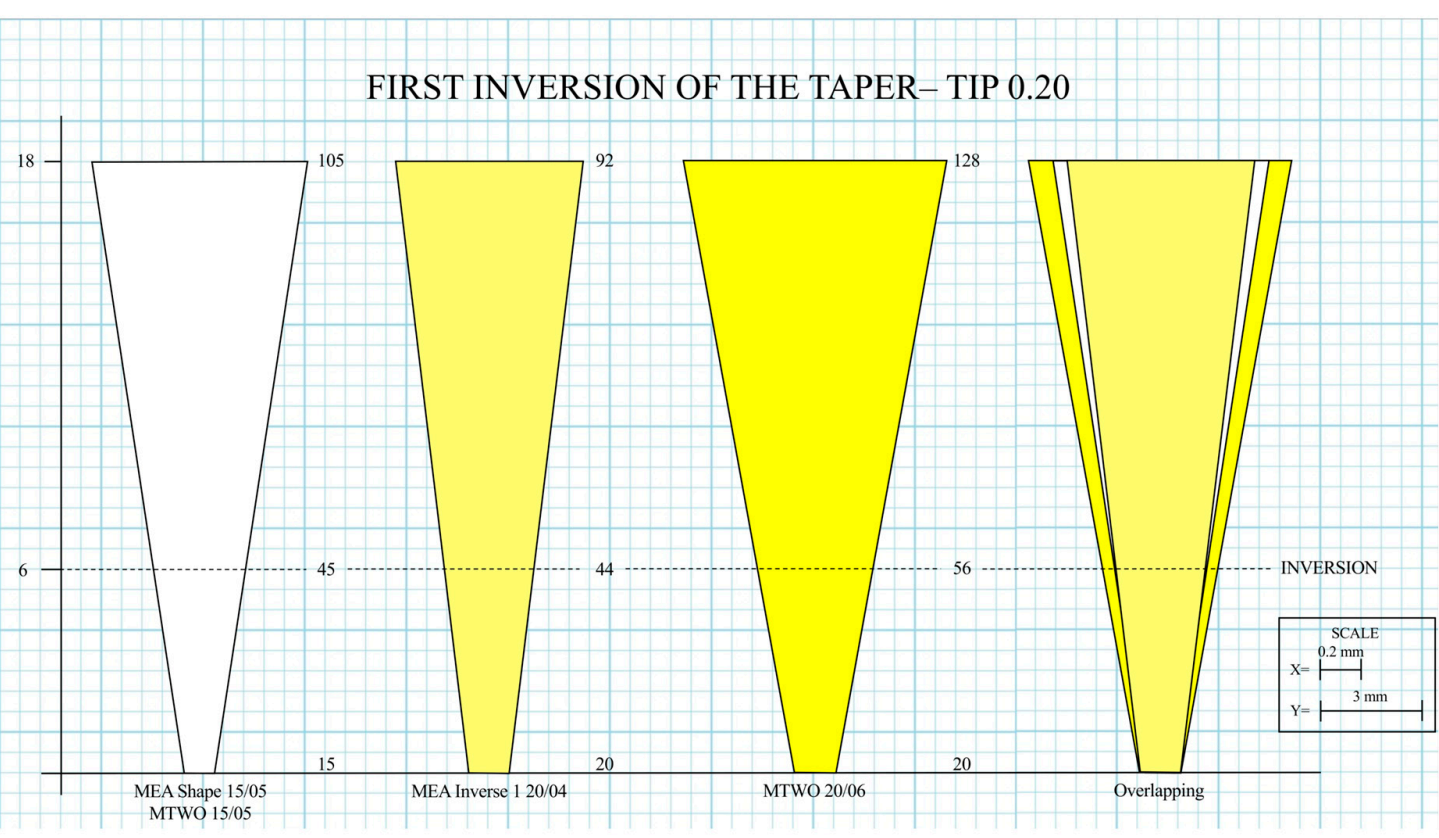
First inversion of the taper at D6 with the Inverse 1 20.04 instrument. Schematic representation showing the initial reduction in canal diameter produced by the transition from the Shape 15.05 file to the Inverse 1 20.04. The localized narrowing at D6 creates the first taper inversion and initiates formation of the coronal retention cavity. The overlapping of the two taper profiles at D6 visually indicates the first point of taper inversion, generating a localized constriction and the onset of the coronal retention cavity.

Detailed geometric analysis of the MEA Inverse Taper^®^ sequence confirmed the presence of two distinct points of taper inversion along the instrumented canal. The first inversion occurred at D6, marking the transition from the Shape 15.05 file to the Inverse 1 20.04 file. The slight reduction in canal diameter (0.45 to 0.44 mm) created a localized constriction that initiated the formation of a coronal retention cavity (Figure 5).

**Figure 6:**
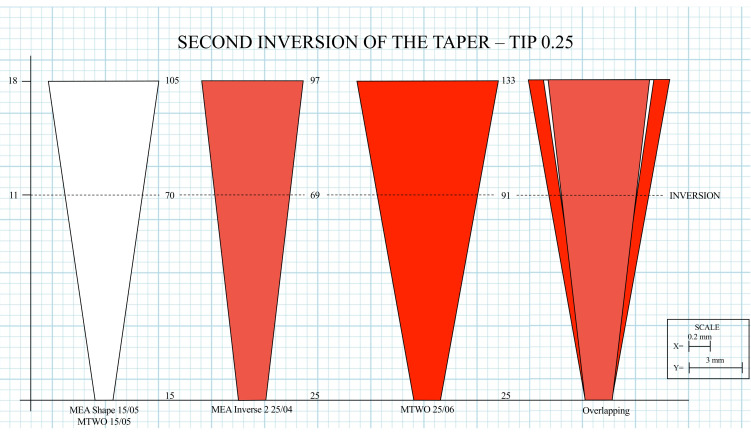
Second inversion of the taper at D11 with the Inverse 2 25.04 instrument. Diagram illustrating the second geometric inversion at D11, where the Inverse 2 25.04 file exhibits a smaller diameter than the preceding Shape 15.05. This second inversion enlarges the retention space coronally and contributes to the continuity of the *RT*. The overlapping of the two taper profiles at D11 highlights the second taper inversion, which extends the retention zone coronally and contributes to the continuity of the RT.

The second inversion was identified at D11, corresponding to the transition from the previously used Shape 15.05 file (0.70 mm) to the subsequent Inverse 2 25.04 file (0.69 mm). This geometric reversal produced a localized reduction in canal diameter, extending the retention zone coronally and contributing to the continuity of the RT (Figure 6).

**Figure 7:**
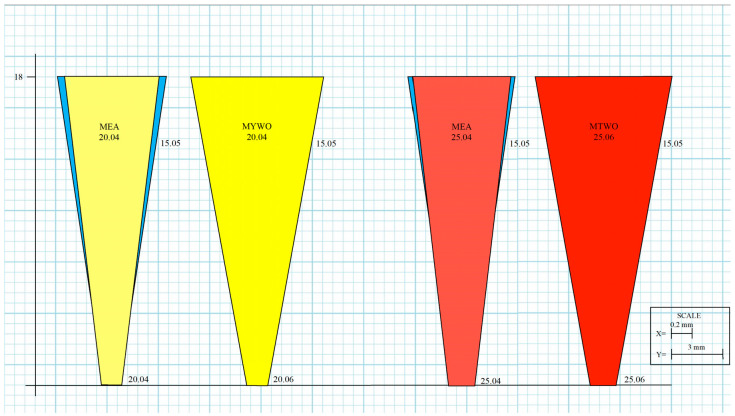
Reconstructed canal geometries obtained after shaping with MEA Inverse Taper^®^ and MTWO systems. Schematic representation of the canal morphology corresponding to instruments 20 and 25, respectively. The color-coded images—yellow (20), red (25)—represent the progressive shaping stages as reconstructed from measured diameters at defined reference levels for the MEA Inverse Taper^®^ and the MTWO system.

To further illustrate these geometric effects, three schematic representations were generated to compare the canal morphology produced by the MEA Inverse Taper^®^ and MTWO systems (Figure 7). The color-coded images (yellow: 20; red: 25) represent progressive shaping stages reconstructed from measured diameters at defined reference levels.

Together, these two inversion points defined a double-taper configuration unique to the MEA Inverse Taper^®^ sequence, providing the structural basis for irrigant retention and fluid stability during shaping.

### 3.4. Fluid Retention and Irrigant Behavior

**Figure 8:**
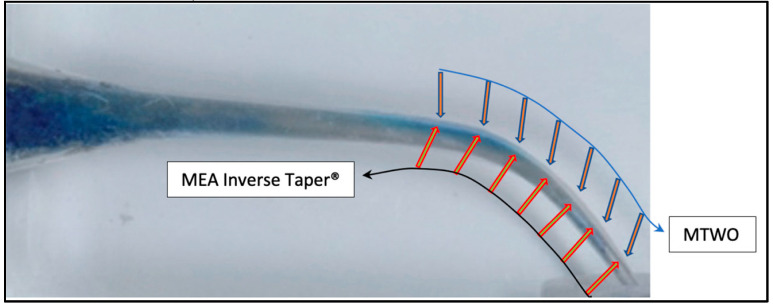
Superimposed images comparing MEA Inverse Taper^®^ and MTWO sequences. Images aligned using reference notches at the coronal and apical ends of the plastic blocks. The MTWO preparation enlarges the canal outward, effectively altering the real initial anatomy. The MEA Inverse Taper^®^ sequence (blue overlay) demonstrates retained irrigant in the coronal third, while MTWO shows complete evacuation. The overlay confirms the presence of the Radicular Tank in the MEA canals.

Quantitative image segmentation of the superimposed photographs demonstrated that the MEA Inverse Taper^®^ sequence retained approximately 35–40% of the initial dye area within the coronal third after final instrumentation, whereas the MTWO system retained less than 5% (Figure 8).

Across all samples, the MEA Inverse Taper^®^ group demonstrated a mean dye-retention area of 38% (range 35–40%), whereas the MTWO group showed a mean retention of approximately 3% (range 2–4%), confirming the markedly different fluid behavior between the two systems. The underlying measurements for each sample are reported in Table 4.

The movement of the irrigant column during shaping substantially differed between systems. In the MTWO canals, irrigant movement showed a characteristic coronal reflux pattern, forming a turbulent return vortex consistent with previously reported flow models [15,16,17].

In contrast, the MEA canals maintained a relatively stable irrigant column, producing a laminar retention layer within the coronal third—effectively functioning as a static “bath” around the rotating file.

These findings indicate that the inverse taper geometry limits fluid extrusion and sustains a constant visual dye volume inside the canal during mechanical shaping. The effect was most pronounced in the coronal and middle thirds, while both systems showed comparable dye removal patterns in the apical region. These observations reflect dye persistence under standardized conditions and do not quantify actual irrigant mass, pressure, or hydrodynamic stability

## 4. Discussion

This proof-of-concept study provides novel experimental evidence demonstrating that an inverse taper geometry can generate a visually detectable intracanal retention phenomenon—termed the Radicular Tank (RT)—during mechanical shaping. The RT should be understood as a functional intracanal dye-retention chamber rather than a new hydrodynamic theory. Its behavior aligns with established models of irrigant residence time and flow stagnation, but its origin is uniquely linked to the geometric inversion produced by the MEA Inverse Taper^®^ technique.

It is important to note that the present study evaluated *dye persistence* rather than actual irrigant volume. The findings should be interpreted as qualitative evidence of the geometric potential for intracanal retention, not as a quantitative demonstration of irrigant mass or pressure dynamics. Future studies incorporating real irrigants and fluid-dynamic measurements will be required to determine whether the observed geometry translates into true irrigant retention.

An important alternative explanation must also be considered. The MEA sequence produces a smaller and more conservative taper than the MTWO system, resulting in reduced instrument–wall contact. Consequently, the higher amount of residual dye observed in the MEA group may partly reflect dye remaining on untouched canal surfaces rather than a true increase in the system’s ability to retain or stabilize fluid. This possibility is inherent to the qualitative nature of the present study and must be acknowledged as a primary interpretive factor. Therefore, dye persistence in this context may represent a combination of geometric taper inversion and decreased dentin engagement, and the current methodology cannot distinguish between these two mechanisms. Future investigations using micro-CT–based wall-contact mapping and quantitative irrigant-flow techniques will be necessary to isolate the contribution of each factor.

In addition to taper geometry, other instrument design features may also influence irrigant behavior. The MEA Inverse Taper^®^ system and the MTWO system differ in cross-sectional profile, flute depth, and cutting-edge configuration. MTWO instruments exhibit an S-shaped cross-section with relatively deep flutes designed to enhance debris removal and cutting efficiency, whereas the MEA system—although also NiTi-based—uses a modified cross-section with more conservative flute engagement to support a minimally invasive shaping strategy. These geometric characteristics may affect chip space, debris transport, and local flow patterns independently of taper inversion. While the present study focused specifically on the hydrodynamic consequences of taper geometry, these additional design differences should be considered when interpreting dye-retention patterns. Future quantitative work, ideally incorporating cross-sectional imaging and flow modeling, will be needed to determine the relative influence of taper, flute geometry, and cross-sectional design on intracanal fluid dynamics.

The results confirm that the MEA Inverse Taper^®^ technique maintains residual dye within the canal lumen, supporting continuous chemo-mechanical preparation under fluid immersion.

The persistence of methylene blue dye throughout shaping with the MEA Inverse Taper^®^ sequence verified that this configuration is capable of producing a persistent residual dye distribution, acting as a qualitative indicator of potential intracanal retention within the coronal and middle thirds of the canal.

From a hydrodynamic perspective, the inverse taper geometry appears to interrupt the typical coronal reflux observed in conventional systems [6,9,11,12,13], stabilizing the irrigant column and promoting laminar flow conditions. Such behavior likely prolongs irrigant residence time, enhancing the chemical interaction between the irrigant and the canal surfaces.

From a mechanical perspective, the reduced wall contact observed between D5 and D10 in the MEA Inverse Taper^®^ sequence may theoretically reduce frictional engagement between the instrument and canal walls. However, this interpretation is based solely on geometric considerations, as the present study did not record torque, friction, or microscopic surface data. Therefore, this observation should be viewed as a preliminary hypothesis rather than a demonstrated mechanical advantage. While previous literature has suggested that limited taper engagement can improve mechanical safety [4,5,23,24], dedicated studies—including SEM analysis and real-time torque or friction measurements—will be required to determine whether the inverse taper geometry truly reduces mechanical stress or instrument fatigue.

Previous studies using computational fluid dynamics (CFD) and in vitro models have shown that standard taper configurations (0.06–0.08) promote apical-to-coronal fluid displacement, leading to stagnation zones and limited irrigant replacement in the apical third [20,23,24]. Even with optimized needle designs or increased taper, such configurations fail to overcome these hydrodynamic limitations [17,20,25].

In contrast, the MEA Inverse Taper^®^ design introduces a controlled geometric reversal that creates a static dye-retention chamber within the canal—representing a fundamental shift in how irrigant motion and residence can be managed during shaping.

The concept of minimally invasive endodontics emphasizes dentin preservation, especially in the pericervical region, to improve the long-term structural integrity of treated teeth [18,19]. While systems with smaller tapers (0.02–0.04) maintain structural integrity, they often reduce canal volume and limit irrigant exchange [26,27,28].

The MEA Inverse Taper^®^ concept integrates both advantages: minimizing dentin removal while promoting residual dye persistence through its reversed geometry. This dual benefit—structural preservation with hydrodynamic enhancement—has not been previously reported in endodontic literature.

The present findings are also consistent with those of Boutsioukis et al. [15] and Versiani et al. [21], who demonstrated that canal shape directly influences irrigant velocity and distribution. The formation of a coronal dye-retention zone, as demonstrated in this study, supports these theoretical models and may explain why certain canal designs produce more effective chemical cleaning despite minimal taper.

Although this study used standardized plastic blocks, the observed RT phenomenon has potential clinical relevance.

Residual dye persistence may theoretically indicate increased irrigant–wall contact—although this reflects only a qualitative visual observation rather than a measurement of true fluid volume—and in other contexts it has been associated with improved tissue dissolution and bacterial reduction [10,26,28].

However, the present study did not evaluate debris removal, cleaning efficacy, or the hauling capacity of the instruments. Therefore, no conclusion can be drawn regarding whether the dye-visualized retention pattern enhances, limits, or has no effect on debris elimination during instrumentation. Dedicated studies will be required to investigate the influence of the Radicular Tank on debris transport and overall cleaning efficiency.

Moreover, maintaining a stable dye-retention environment during instrumentation creates an ‘instrumentation-in-bath’ condition, in which the rotating file operates continuously within a liquid medium. This configuration may influence factors such as debris transport, frictional heat generation, or chemical interaction; however, these potential effects remain theoretical in the absence of direct measurements. The present study did not assess debris removal or cleaning efficacy, and therefore, no conclusions can be drawn regarding whether this qualitatively observed dye-retention phenomenon enhances or limits mechanical or chemical performance. These aspects warrant dedicated quantitative investigation using torque sensors, thermography, SEM, and real-time CFD analysis [12,13]

The MEA Inverse Taper^®^ geometry also aligns with the current trend toward conservative endodontics. By avoiding excessive coronal enlargement, the system reduces unnecessary dentin removal and preserves canal curvature, potentially improving fracture resistance and restorative outcomes [18,19,23,24]. Although fracture resistance was not measured in this study, the observed geometric characteristics suggest potential biomechanical benefits that merit quantitative evaluation in future research.

The major strength of this study lies in its standardized, reproducible design, which enabled clear visualization and qualitative validation of the RT. Nevertheless, some limitations must be acknowledged. The use of plastic endodontic blocks, while allowing high reproducibility and visualization, does not reproduce the physical or chemical properties of natural dentin —including permeability, elasticity, frictional behavior, and irrigant absorption—which are critical for realistic hydrodynamic and mechanical analysis. These standardized transparent blocks were intentionally selected to isolate the geometric effects of taper inversion without anatomical variability, but validation in natural dentin models will be essential to confirm biological and mechanical behavior. Therefore, the RT phenomenon described here should be interpreted as an experimental validation rather than direct clinical evidence. Although methylene blue provided excellent contrast for visualizing irrigant retention, it does not reproduce the physicochemical properties of sodium hypochlorite or EDTA—such as surface tension, viscosity, and reactivity—which are crucial for determining irrigant dynamics under clinical conditions. Future studies should therefore incorporate real irrigants to assess whether these properties influence the formation and stability of the Radicular Tank.

An additional limitation is that the two shaping systems were not matched for final taper: the MEA sequence ended at 25/0.04, whereas MTWO ended at 25/0.06. This difference may inherently affect dye displacement and retention, therefore limiting any direct “performance” comparison between systems. Accordingly, the present findings should be interpreted as qualitative feasibility evidence that a controlled taper inversion can generate a dye-retention phenomenon, rather than as definitive proof of superiority. Future studies will include comparators matched for apical size and taper and will incorporate quantitative hydrodynamic measurements.

Another limitation of the present study is that irrigant behavior and mechanical effects were assessed predominantly through visual (photographic) analysis, without quantitative measurements of fluid dynamics, torque, or intracanal pressure. This qualitative approach reflects the proof-of-concept nature of the study, but comprehensive quantitative characterization remains essential and is outlined in the subsequent section.

A further limitation is the comparison with only one conventional shaping system (MTWO). Although MTWO is a well-established continuous-taper reference system that provides a meaningful contrast to the inverse-taper geometry, using a single comparator inevitably restricts the generalizability of the findings. Future studies should therefore include shaping systems with different taper geometries, metallurgies, and kinematics to evaluate whether the RT effect is unique to the inverse taper design or may occur, to varying degrees, in other shaping philosophies. These considerations reinforce the need for expanded experimental and computational investigations.

Future investigations should further develop this concept through multi-scale modeling and clinical validation. Computational fluid dynamics (CFD) simulations are needed to characterize pressure gradients, flow velocities, and shear stress distribution within the RT zone, while micro-CT and three-dimensional morphometric analyses of extracted teeth should confirm taper inversion and fluid retention under realistic dentin conditions. In addition, in vitro and in vivo studies comparing bacterial reduction, debris removal, and torque behavior between inverse and conventional taper systems will be essential to establish the clinical relevance of this geometry. Demonstrating that the RT can be consistently reproduced under clinical conditions would represent a major advancement in the understanding of endodontic hydrodynamics and could redefine current principles of canal shaping and irrigation.

## 5. Conclusions

Despite the limitations of this proof-of-concept study, several conclusions can be drawn. The MEA Inverse Taper^®^ technique produced a controlled inversion of taper that enabled dye persistence within the root canal during mechanical shaping. This retained volume, defined as the Radicular Tank, represents a distinct and reproducible visual intracanal retention phenomenon that prolongs irrigant residence time and stabilizes intracanal fluid behavior. Compared with a conventional system (MTWO), the inverse taper design generated less coronal displacement of dye, greater dye stability, and a more conservative shaping geometry, preserving canal curvature and pericervical dentin. The presence of the RT may enhance the efficiency of chemo-mechanical preparation by maintaining continuous dye presence along the canal walls throughout instrumentation. Further quantitative and clinical investigations—including computational fluid dynamics, micro-CT, and torque measurement analyses—are required to validate these findings and determine their applicability to natural root canal systems. Demonstrating reproducible RT formation under clinical conditions would represent a significant advancement in understanding endodontic hydrodynamics and could redefine future shaping and irrigation strategies.

## Figures and Tables

**Table 1:** Materials and equipment used in the study.

Item	Product	Manufacturer	Location
Shaping system	MEA^®^ Adrenaline (MEA Inverse Taper^®^ sequence)	Shenzhen Perfect Medical Instrument Co.	Shenzhen, China
Shaping system	MTWO	VDW GmbH	Munich (Germany)
Endodontic motor	MotoSync Pro	Xpedent	Guilin, China
Irrigant tracer	Methylene blue 1%	Sigma-Aldrich	St. Louis, MO, USA
K-file	ISO #15 K-File (15.02)	Shenzhen Perfect Medical Instrument Co.	Shenzhen, China
Microscope	Univet 6×	Univet	Rezzato, Italy
LED light	Zumax Medical	Zumax Medical Co., Ltd.	Suzhou, China
Camera	Nikon D3000	Nikon Corp.	Tokyo, Japan
Tripod	Manfrotto	Manfrotto	Cassola, Italy
Software	Adobe Photoshop CS6	Adobe Systems	San Jose, CA, USA

**Table 2:** MEA Inverse Taper^®^ Sequence. Canal diameters measured at different canal levels (D0–D11) according to the instrument used (Shape 15.05, Inverse 1 20.04 and Inverse 2 25.04).

D (mm)	Shape 15.05	Inverse 1 20.04	Inverse 2 25.04
11	0.70	0.64	0.69
10	0.65	0.60	0.65
9	0.60	0.56	0.61
8	0.55	0.52	0.57
7	0.50	0.48	0.53
6	0.45	0.44	0.49
5	0.40	0.40	0.45
4	0.35	0.36	0.41
3	0.30	0.32	0.37
2	0.25	0.28	0.33
1	0.20	0.24	0.29
0	0.15	0.20	0.25

**Table 3:** MTWO Sequence. Canal diameters measured at different canal levels (D0-D11) according to the instrument used (15.05, 20.06, and 25.06).

D (mm)	15.05	20.06	25.06
11	0.70	0.86	0.91
10	0.65	0.80	0.85
9	0.60	0.74	0.79
8	0.55	0.68	0.73
7	0.50	0.62	0.67
6	0.45	0.56	0.61
5	0.40	0.50	0.55
4	0.35	0.44	0.49
3	0.30	0.38	0.43
2	0.25	0.32	0.37
1	0.20	0.26	0.31
0	0.15	0.20	0.25

**Table 4:** Residual dye-retention percentages (%) for all samples in each experimental group. Values were obtained through pixel-based image segmentation as described in the Methods. The MEA Inverse Taper^®^ sequence showed higher residual-dye persistence (mean 38%, range 35–40%), whereas the MTWO system showed minimal dye retention (mean 3%, range 2–4%).

Specimen	MEA Inverse Taper^®^ (%)	MTWO (%)
1	35	2
2	38	3
3	40	4

## Data Availability

The original contributions presented in this study are included in the article. Further inquiries can be directed to the corresponding author.
